# Prenatal circulating microRNA signatures of foetal Down syndrome

**DOI:** 10.1038/s41598-018-35876-5

**Published:** 2019-02-20

**Authors:** Monika Zbucka-Kretowska, Magdalena Niemira, Magdalena Paczkowska-Abdulsalam, Agnieszka Bielska, Anna Szalkowska, Ewa Parfieniuk, Michal Ciborowski, Slawomir Wolczynski, Adam Kretowski

**Affiliations:** 1 Department of Reproduction and Gynaecological Endocrinology, Medical University of Bialystok, Bialystok, Poland; 2 Clinical Research Centre, Medical University of Bialystok, Bialystok, Poland; 3 Clinical Research Centre; Department of Endocrinology, Diabetology and Internal Medicine, Medical University of Bialystok, Bialystok, Poland

## Abstract

The altered expression pattern of miRNAs might potentially reflect anomalies related to foetal chromosomal aberrations. The aim of the study was to determine the expression level of miRNAs in plasma of pregnant women with foetal Down syndrome (DS). Out of 198 amniocentesis performed at 15–18 weeks of gestation, within a group of 12 patients with foetal DS and 12 patients with uncomplicated pregnancies, who delivered healthy newborns at term, we examined the expression level of 800 miRNAs using the NanoString technology. Our study revealed that there are 6 miRNAs were upregulated (hsa-miR-15a, hsa-let-7d, hsa-miR-142, hsa-miR-23a, hsa-miR-199, hsa-miR-191) and 7 were downregulated (hsa-miR-1290, hsa-miR-1915, hsa-miR30e, hsa-miR-1260, hsa-miR-483, hsa-miR-548, hsa-miR-590) in plasma samples of women with foetal DS syndrome. The genes regulated by identified miRNAs are involved in central nervous system development, congenital abnormalities and heart defects. The results of the present study yielded information on DS-specific miRNA expression signature, which can further help to design a panel of miRNAs as a non-invasive test for DS diagnosis. We believe that identified miRNAs may attend in the pathogenesis of DS and would potentially make a significant role for the future preventive therapies.

## Introduction

Down syndrome (DS), known as the most frequent chromosomal abnormality, is caused by an extra chromosome 21 or a fragment thereof^1^. According to the National Institute of Child Health & Human Development (http://www.ncbi.nlm.nih.gov/pubmed/) the incidence of DS in the United States is estimated to be 1:800-1:1.000 live births. This trisomy concerns congenital anomalies, including heart defects, gastrointestinal anomalies, immune system defects, thyroid disease, bone defects, genitourinary system defects, intellectual disability and many other diseases^2^. Currently, prenatal diagnosis of DS is based on invasive techniques that verify the occurrence of chromosomal aberration, while non-invasive methods are used to estimate the risk. Since amniocentesis as an invasive approach is associated with 1% risk of foetal damage or miscarriage^3^, the focus of research community has been placed recently on the discovery of biomarkers, allowing for a non-invasive foetal DS diagnosis. A significant breakthrough in this field was made, when genetic testing of free foetal DNA (ffDNA), circulating in maternal peripheral blood, was developed and brought to the market. However, despite considerably low false positive rates for DS screening (0.5%), these diagnostic kits are still highly priced^4–8^. All mentioned prenatal diagnostic methods are focused on cytogenetic confirmation of the abnormal karyotype. In fact, we have to realize that, on the one hand, the abnormal foetal karyotype can be diagnosed in cytogenetic studies, but on the other hand, we can potentially find some biochemical variations in maternal plasma, which appeared due to stimulation by the extra chromosome 21.

MicroRNAs (miRNAs) form a large group of small non-protein coding RNAs, which are considered important regulators of gene expression^9^. The miRNA genes are located in both coding and non-coding regions of the genome. They exert their regulatory function through the interaction of their “seed” sequences with 3′-end, and more rarely with 5′-end, of mRNA transcribed from target genes, followed by degradation of target mRNA. The reduction in the amount of a specific mRNA is an important result of this molecular event. MiRNAs have diverse expression patterns and might regulate various developmental and physiological processes^10,11^. These short RNAs have been implicated in the widespread control of critical biological processes such as proliferation, differentiation, and apoptosis. Moreover, abnormal miRNA expression is associated with the development of various diseases, such as cancer, cardiovascular disease, metabolic disorders, intellectual disability, and trisomy 21.

Since miRNA plays a pivotal role in the epigenetic regulation of mRNA expression, it has become a target of research directed towards the development of a new non-invasive method for foetal DS screening. The diversity of miRNAs expression in trisomy 21 was already reported, but the published data are not consistent^12^. Up to date, alterations in miRNAs expression in DS have been investigated focusing mainly on miRNAs derived from human chromosome 21. On the other hand, though, taking into consideration the complexity of epigenetic gene expression regulation, miRNAs derived from chromosomes other than chromosome 21, are likely to be involved as well. It has been previously reported that miRNA can regulate a large number of protein-coding genes, and multiple miRNAs can regulate a single target gene^13^. Therefore, we decided to use the NanoString technology to evaluate expression pattern of 800 miRNA molecules encoded on different chromosomes. nCounter miRNA panel content is based on miRBase, a bioinformatics repository for small RNA sequence and annotation information^14^. The results of our analysis could yield information on miRNA expression patterns specific for DS. Our study might not only identify molecules potentially involved in disorder pathogenesis, but most importantly help to design a panel for non-invasive DS screening.

## Results

### Identification of a serum miRNA profile for foetal Down syndrome

To identify miRNAs with potential diagnostic value, we performed miRNA profiling of plasma from DS patients (n = 12) and healthy control subjects (n = 12), using the NanoString Technology platform. The clinical characteristics of all participants is presented in Table 1. The group of 13 miRNAs with the most significant differences in expression between the two compared groups (p < 0.05 = the unadjusted *p* value) was selected using a t test. Specifically, 6 miRNAs were upregulated (hsa-miR-15a, hsa-let-7d, hsa-miR-142, hsa-miR-23a, hsa-miR-199, hsa-miR-191) and 7 downregulated (hsa-miR-1290, hsa-miR-1915, hsa-miR30e, hsa-miR-1260, hsa-miR-483, hsa-miR-548, hsa-miR-590) in plasma samples of DS patients versus healthy control subjects (Table 2).

**Table 1:** Clinical characteristics of the patients.

	Down Syndrome Pregnancies (n = 12)	Pregnancies without Down Syndrome (n = 12)	*p* value
Maternal age (mean ± SD)	38,5 ± 2,95	38 ± 4,17	0,17
Body mass index (kg/m^2^) (mean ± SD)	23,75 ± 2,65	23,58 ± 3,32	0,46
Gestational age (mean ± SD)	16 ± 1,07	17 ± 1,19	0,54

SD - standard deviation.

The *p* value describes significance of difference between women with foetal Down syndrome (DS) in comparison to healthy controls using Mann-Whitney U-test.

**Table 2:** miRNAs upregulated and downregulated in DS group.

miRNA ID	Counts in DS group	Counts in control group	Fold change	*p* value	Genome context
hsa-miR-15a-5p	42,28 ± 13,63	23,81 ± 15,35	**1**,**78 ↑**	0,0013	Chr13
hsa-let-7d-5p	115,26 ± 91,69	66,72 ± 41,87	**1**,**73 ↑**	0,0226	Chr9
hsa-miR-142-3p	185,16 ± 84,58	114,81 ± 107,95	**1**,**61 ↑**	0,0349	Chr17
hsa-miR-23a-3p	322,96 ± 180,07	198,01 ± 67,98	**1**,**63 ↑**	0,0371	Chr19
hsa-miR-199a-3p	214,61 ± 51,64	135,35 ± 39,56	**1**,**59 ↑**	0,0431	Chr19
hsa-miR-191-5p	235,33 ± 205,31	123,13 ± 81,62	**1**,**91 ↑**	0,0481	Chr3
hsa-miR-1290	53,27 ± 18,12	95,84 ± 40,14	**1**,**81 ↓**	0,0018	Chr1
hsa-miR-1915-3p	10,63 ± 6,89	19,69 ± 7,83	**1**,**85 ↓**	0,0068	Chr10
hsa-miR-30e-5p	49,37 ± 10,94	62,96 ± 12,17	**1**,**28 ↓**	0,0097	Chr1
hsa-miR-1260a	11,05 ± 4,26	15,84 ± 5,85	**1**,**43 ↓**	0,026	Chr14
hsa-miR-483-3p	14,44 ± 5,14	19,35 ± 5,8	**1**,**34 ↓**	0,031	Chr11
hsa-miR-548n	29,97 ± 10,31	45,15 ± 13,05	**1**,**52 ↓**	0,043	Chr7
hsa-miR-590-5p	19,63 ± 7,99	28 ± 10,5	**1**,**43 ↓**	0,046	Chr7

The *p* value describes significance of difference between women with foetal Down syndrome (DS) in comparison to healthy controls using nSolver Software; miRNA ID = official microRNA name according to miRBASE; *p* value = the unadjusted *p* value from the t test.

### Putative targets genes for miRNAs

**Figure 1:**
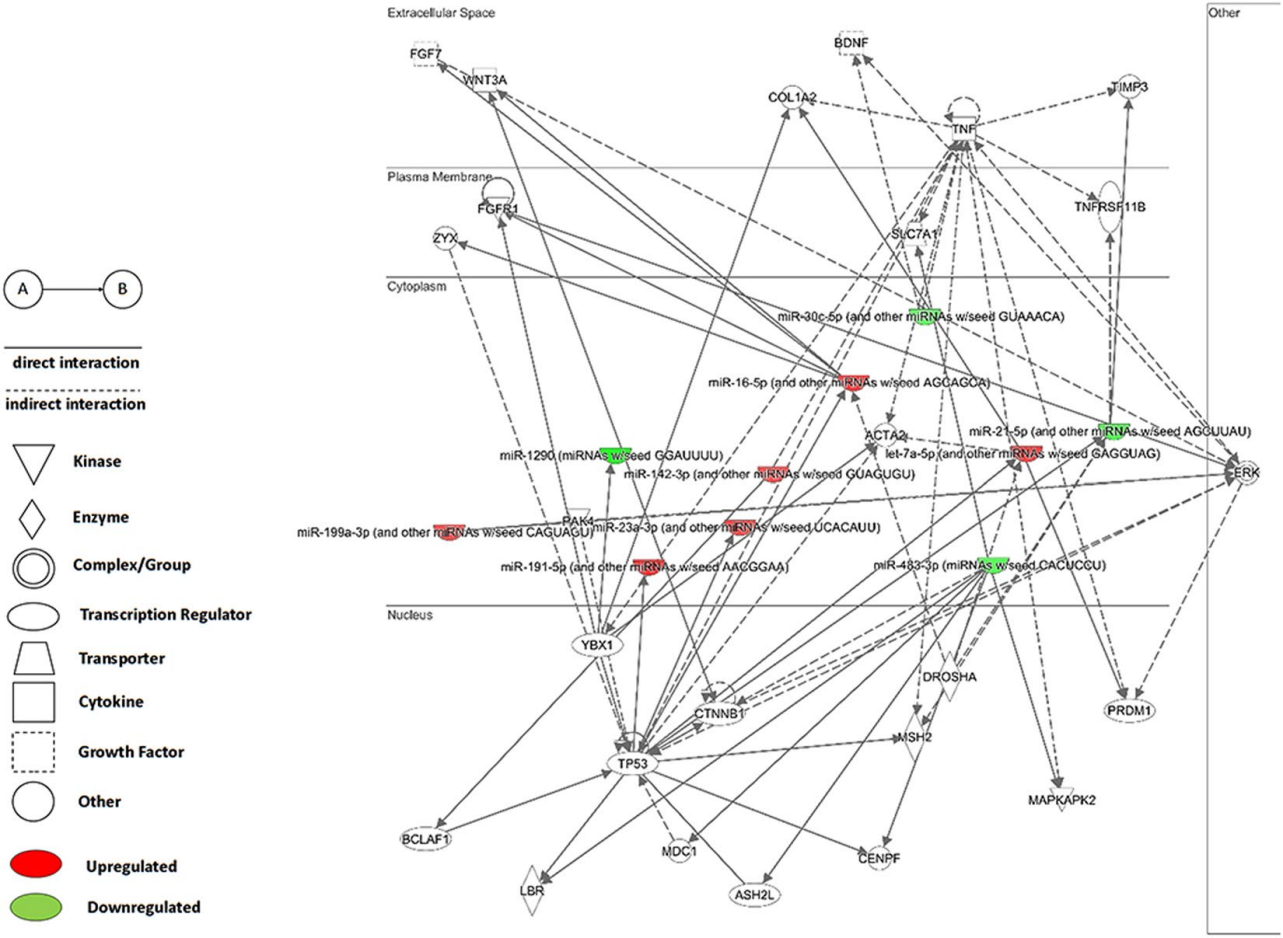
The highest scoring miRNA-mRNA interaction network were generated through the use of IPA (QIAGEN Inc., https://www.qiagenbio-informatics.com/products/ingenuity-pathway-analysis).

To examine the function of miRNAs identified as potential biomarkers for DS, we revealed putative target genes for each of the differentially expressed molecules using Ingenuity Pathway Analysis, a tool for analysis, integration and interpretation of data derived from ‘omics’ experiments. That powerful data integration allows to uncover the significance of data and identify new targets or create the interactive network within the context of biological systems^15^. Based on this tool we proposed a new integrative network including target genes and miRNAs (Fig. 1). The genes selected as potentially regulated by selected miRNAs were divided into 7 functional clusters using DAVID Functional Annotation Clustering Tool integrated in DAVID Bioinformatics Resources^16^. Disease association analysis was performed using IPA. Cancer pathway were identified as the most highly affected, then pathway connected with reproductive system disease, organismal injury and abnormalities, hematological disease and immunological processes.

## Discussion

The growing number of studies examining the global changes in miRNA expression suggest that these molecules are associated with a variety of human diseases, such as neuropsychiatric disorders^17^, diabetes^18^ and cancer^19^. The expression levels of biofluids–derived miRNAs represent potential novel tools for diagnostic and disease activity assessment purpose^20^. Although several technological options are available to analyse miRNA expression comprehensively, the detection of miRNAs in and subsequent interpretation of such data can be strongly influenced by the specific platforms used. In our study, we focused on a comparison of miRNA expression profiles between maternal plasma samples from pregnant women with euploid or T21 foetuses. We hypothesized that the plasma-derived miRNAs could potentially represent novel means for the prenatal non-invasive diagnosis of foetal Down syndrome. To our best knowledge, this study is the first miRNA profiling in plasma maternal sample based on NanoString nCounter platform. Above platform has been shown in comparison test to detect miRNAs in biofluids with greater sensitivity and specificity than other miRNA detection methods and offers high quality data due to the limited RNA amplification that could potentially introduce bias toward the most abundant miRNAs^21^.

Our study revealed that 13 miRNAs are differentially expressed in women with foetal Down syndrome in comparison to healthy donors. Despite selected miRNAs were exceed FDR-corrected criterion, we believe that the candidates miRNAs have a high probability of being specific for plasma pregnant women with T21 foetuses. Previous study have shown the involvement of five chromosome 21- derived miRNAs using real-time PCR assay and they observed no differences between pregnant women with euploid or Down syndrome foetuses^22^.

Erturk *et al*. evaluated 14 chromosome 21-derived miRNAs in maternal plasma and found increased levels of miR-99a and miR-3156, when compared to patients carrying euploid foetuses^23^. Lim *et al*. reported altered expression of placenta – specific miRNAs, miR – 1973 and miR – 3196 were overexpressed in trisomy 21 placenta. These two miRNAs may regulate target genes involved in development of the nervous system^24^. This data suggests that miRNA profiling may be a promising strategy in the development of non-invasive prenatal testing. Moreover, researchers highlighted the importance of the assessment of other miRNAs related to chromosome 21.

The expression levels of miRNAs were also investigated in DS individuals. Xu *et al*. identified overexpressed miR-99a in lymphocytes, linking this observation to immunological defects in trisomy 21^12^. The investigators focused on searching for the differences in miRNA expression in villus samples. However, they analysed only maternal blood of patients with euploid foetus, but not with foetal DS. Shi *et al*.^25^ studied the miRNA expression profile of hippocampal tissue from DS foetuses using miRNA microarray, and reported that the function of miR-138-5p and the downregulation of its target, enhancer of zeste homolog 2, in the hippocampus may be involved in the intellectual disability of DS patients. A pregnancy with a DS foetus is accompanied by a great number of biochemical variations in maternal plasma, probably induced by the additional chromosome 21. The latest reports show that pregnancies with foetal chromosomal aberrations are strongly connected with imbalance in chemokines and bioactive lipids, such as sphingolipids. It indicates a new potential biological mechanism of foetal DS^26–30^.

We hypothesize that the additional foetal chromosome 21 could potentially have an impact on the expression of miRNAs encoded on different pairs of chromosomes as well.

Since the pathology of the syndrome is extremely complicated and the additional chromosome 21 causes multiple foetal pathologies and induces maternal immune response, searching for abnormal signals and understanding the pathological mechanism are highly desired. For that purpose, differentially expressed miRNA molecules were analysed using bioinformatics tools to explore their biological function (Fig. 1). As shown Table 3, the target genes regulated by miRNAs characterised in T21 are involved in the central nervous system development, congenital abnormalities, heart defects.

**Table 3:** Functional annotation clustering according to DAVID Functional Annotation Clustering Tool integrated in DAVID Bioinformatics Resources.

Gene cluster 1	
TIA1	TIA1 cytotoxic granule associated RNA binding protein
RBMS1	RNA binding motif single stranded interacting protein 1
IGF2BP3	Insulin like growth factor 2 mRNA binding protein
NCL	Nucleolin
HNRNPA0	Heterogenous nuclear ribonucleoprotein A0
RBM19	RNA binding motif protein 19
**Gene cluster 2**	
MAP4K4	Mitogen-activated protein kinase 4
MAPKAPK2	Mitogen-activated protein kinase-activated protein kinase 2
WEE1	WEE1 G2 checkpoint kinase
BMP2K	BMP2 inducible kinase
HIPK3	Homeodomain interacting protein kinase
CHEK1	Checkpoint kinase 1
**Gene cluster 3**	
KRT85	Keratin 85
KRT19	Keratin 19
LMNB1	Lamin B1
VIM	Vimentin
KRT7	Keratin 7
LMNB2	Lamin B2
**Gene cluster 4**	
RAB21	Member RAS oncogene family
RHOG	Ras homolog family member
RAB9B	Member RAS oncogene family
RHOB	Ras homolog family member
RAB30	Member RAS oncogene family
RAB27B	Member RAS oncogene family
**Gene cluster 5**	
CDCP1	CUB domain containing protein 1
SLC16A10	Solute carrier family 16 member 10
SLC7A11	Solute carrier family 7 member 11
SLC38A1	Solute carrier family 38 member 1
SLC7A1	Solute carrier family 7 member 1
SLC38A5	Solute carrier family 38 member 5
SLC38A2	Solute carrier family 38 member 2
**Gene cluster 6**	
SLC25A1	Solute carrier family 25 member 1
SLC25A32	Solute carrier family 25 member 32
SLC25A24	Solute carrier family 25 member 24
UCP2	Uncoupling protein 2
SLC25A13	Solute carrier family 25 member 13
SLC25A22	Solute carrier family 25 member 22
**Gene cluster 7**	
CLDN12	Claudin 12
TMEM251	Transmembrane protein 251
C2orf74	Chromosome 2 open reading frame 74
C17orf80	Chromosome 17 open reading frame 80
TVP23B	Trans-golgi network vesicle protein 23 homolog B
TMEM59	Transmembrane protein 59
RFT1	RFT1 homolog
CA12	Carbonic anhydrase 12
TMEM87A	Transmembrane protein 87A
TMEM106C	Transmembrane protein 106C
SLC8A5	Solute carrier family 8 member 5
TMEM41B	Transmembrane protein 41B
NIPAL2	NIPA like domain containing 2
FAM69A	Family with sequence similarity 69 member A
CDCP1	CUB domain containing
ACP2	Acid phosphatase 2, lysosomal
SLC35B3	Solute carrier family 35 member B3
PRRG4	Proline rich and Gla domain 4
YIF1B	Yip1 interacting factor homolog B, membrane trafficking protein

Our results provide a new perspective for the pathomechanism of the foetal trisomy 21 and a novel direction for future research of molecular pathways involved in the disorder. However, these data should be viewed as preliminary, we believe that it opens the new possibility that the miRNAs are novel noninvasive biomarkers. The expression patterns of miRNAs in Down syndrome pregnancies may have the crucial role for further improvement of prenatal screening.

## Methods

### Study subjects

The study and control groups consisted of 198 women (mean age 33.4 yrs, range: 17–45 yrs) who underwent routine amniocentesis between the 15^th^ and 18^th^ weeks of gestation at the Department of Reproduction and Gynaecological Endocrinology of the Medical University of Bialystok, Poland. Participants received detailed information on the study and associated risks prior to enrolment. Maternal serum samples were collected directly after amniocentesis, centrifuged and stored at −80 °C. After chromosomal analyses, 12 patients with foetal trisomy 21 and 12 women (Table 1) with foetal normal karyotype were qualified for the study. Women with any pathology of pregnancy, such as hypertension, diabetes or inflammatory diseases, were excluded. All study participants provided written informed consent and received detailed information on the study and associated risks prior to enrolment. The methods were carried out in accordance with the approved guidelines and was conducted in accordance with the ethical standards of the institutional research committee, with the 1964 Helsinki declaration and was approved by the local ethics committee of the Medical University of Bialystok, Poland (approval number: R-I-002/36/2014).

### Detection of miRNA profile

miRNAs were isolated from serum samples using the miRNeasy Serum/Plasma Kit (Qiagen, Valencia, CA) according to the manufacturer’s instructions. The samples were prepared for nCounter miRNA expression profiling according to the manufacturer’s recommendations (NanoString). Briefly, 3 ng miRNA samples were prepared by ligating a specific miR-tag onto 3′ end of each mature miRNA followed by an overnight hybridization (65 °C) to nCounter Reporter and Capture probes. Subsequently, samples were placed into the nCounter Prep Station for automated sample purification and subsequent reporter capture. Each sample was scanned on the nCounter Digital Analyzer for data collection.

### Data Analysis

Statistical analyses were performed with the Statistica software (version 13.1). All data were presented as means ± SD. Mann-Whitney *U* test was used to examine the statistical difference in clinical parameters between samples derived from healthy control subjects and patients with DS foetuses. nSolver 4.0 Analysis software (NanoString) was used for data analysis including normalization using the average geometric mean of the top 100 probes detected. T test with Benjamini-Hochberg false discovery rate correction for multiple comparison were used to determine differential miRNA expression.

### miRNA targets prediction and functional annotation of the selected miRNA targets

To examine the functions of miRNAs identified, miRNA target prediction was performed using Ingenuity Pathway Analysis (QIAGEN Inc., https://www.qiagenbioinformatics.com/products/ingenuity- pathway-analysis). In addition, IPA was used to create the interactive network based on the selected genes list and miRNAs. Functional annotation clustering was carried out using DAVID 6.8 (Database for Annotation, Visualization and Integrated Discovery, https://david.ncifcrf.gov/). The DAVID analysis allows to associate the given gene list with specific functional annotations, which are further divided into the functional clusters listed according to an enrichment *p* value. The Bonferroni correction was used to control the false discovery rate.

## Data Availability

The datasets generated and analysed during the current study are available from the corresponding authors on reasonable request.
